# Planning With Patient-Specific Rectal Sub-Region Constraints Decreases Probability of Toxicity in Prostate Cancer Radiotherapy

**DOI:** 10.3389/fonc.2020.01597

**Published:** 2020-09-11

**Authors:** Caroline Lafond, Anaïs Barateau, Joël N'Guessan, Nicolas Perichon, Nolwenn Delaby, Antoine Simon, Pascal Haigron, Eugenia Mylona, Oscar Acosta, Renaud de Crevoisier

**Affiliations:** Univ Rennes, CLCC Eugène Marquis, INSERM, LTSI - UMR 1099, Rennes, France

**Keywords:** prostate cancer radiotherapy, rectal bleeding, toxicity, plan optimization, organ-at-risk sparing, voxel-wise analysis

## Abstract

**Background:** A rectal sub-region (SRR) has been previously identified by voxel-wise analysis in the inferior-anterior part of the rectum as highly predictive of rectal bleeding (RB) in prostate cancer radiotherapy. Translating the SRR to patient-specific radiotherapy planning is challenging as new constraints have to be defined. A recent geometry-based model proposed to optimize the planning by determining the achievable mean doses (AMDs) to the organs at risk (OARs), taking into account the overlap between the planning target volume (PTV) and OAR. The aim of this study was to quantify the SRR dose sparing by using the AMD model in the planning, while preserving the dose to the prostate.

**Material and Methods:** Three-dimensional volumetric modulated arc therapy (VMAT) planning dose distributions for 60 patients were computed following four different strategies, delivering 78 Gy to the prostate, while meeting the genitourinary group dose constraints to the OAR: (i) a standard plan corresponding to the standard practice for rectum sparing (STD_pl_), (ii) a plan adding constraints to SRR (SRR_pl_), (iii) a plan using the AMD model applied to the rectum only (AMD_RECT_pl_), and (iv) a final plan using the AMD model applied to both the rectum and the SRR (AMD_RECT_SRR_pl_). After PTV dose normalization, plans were compared with regard to dose distributions, quality, and estimated risk of RB using a normal tissue complication probability model.

**Results:** AMD_RECT_SRR_pl_ showed the largest SRR dose sparing, with significant mean dose reductions of 7.7, 3, and 2.3 Gy, with respect to the STD_pl_, SRR_pl_, and AMD_RECT_pl_, respectively. AMD_RECT_SRR_pl_ also decreased the mean rectal dose by 3.6 Gy relative to STD_pl_ and by 3.3 Gy relative to SRR_pl_. The absolute risk of grade ≥1 RB decreased from 22.8% using STD_pl_ planning to 17.6% using AMD_RECT_SRR_pl_ considering SRR volume. AMD_RECT_SRR_pl_ plans, however, showed slightly less dose homogeneity and significant increase of the number of monitor units, compared to the three other strategies.

**Conclusion:** Compared to a standard prostate planning, applying dose constraints to a patient-specific SRR by using the achievable mean dose model decreased the mean dose by 7.7 Gy to the SRR and may decrease the relative risk of RB by 22%.

## Introduction

Rectal toxicity is one of the main side effects arising when treating prostate cancer with radiotherapy. Five-year grade ≥1 and ≥2 rectal bleeding (RB) rates have been reported to be around 30 and 10%, respectively, when combining intensity-modulated radiation therapy (IMRT) with image-guided radiotherapy (IGRT) (1, 2). Several strategies may be implemented in order to spare the rectum and therefore decrease toxicity. For instance, by increasing mechanically the anterior perirectal space, using hydrogel spacer, has been shown to significantly reduce rectal irradiation (3–5). Such an approach is, however, invasive and expensive. A more appealing approach, in the context of dose escalation, would be to intervene at the planning step by adding dosimetric constraints to particular portions of the rectum which may be highly radiosensitive. This requires, however, a robust technique for identifying patient-specific rectal sub-regions (SRR) that should be spared in the treatment planning.

In response to this question, voxel-based methods have already been applied for unveiling spatially variable dose–effect patterns, thereby allowing the identification of sub-regions at risk in several anatomical locations such as the lungs (6), the heart (7), head and neck (H&N) (8), and the bladder (9). Overall, the principles of voxel-based methods rely on the analysis of the local dose–toxicity relationship at fine spatial scales, through (i) non-rigid registration (8), (ii) dose resampling to a common space, and (iii) voxel-wise comparisons between patients with and without toxicity (10, 11). The methodology and pitfalls of voxel-wise analysis are discussed in detail in (12). With respect to rectal toxicity in case of prostate IMRT, a sub-region in the inferoanterior hemianorectum, which will be considered in this study as SRR, was identified by voxel-based analysis as highly predictive of RB (10).

Once a sub-region is identified as predictive for toxicity, the addition of dosimetric constraints to be applied during the planning is, however, challenging. There are no specific recommendations on dose optimization for these original sub-regions as they can be considered as independent structures at risk. A possible strategy to solve this issue is the application of the model proposed by Moore et al. (13) aimed to determine, at the inverse planning step, an achievable mean dose (AMD) in the organs at risk (OARs). Indeed, they showed that, compared to standard dose volume constraints, the mean dose in various OARs could be decreased by using a geometry-based population model relying on volume overlap between the planning target volume (PTV) and OAR. The concept of AMD can be extended to a specific sub-region to be spared (instead of considering the whole OAR) while preserving target coverage.

**Figure 1:**
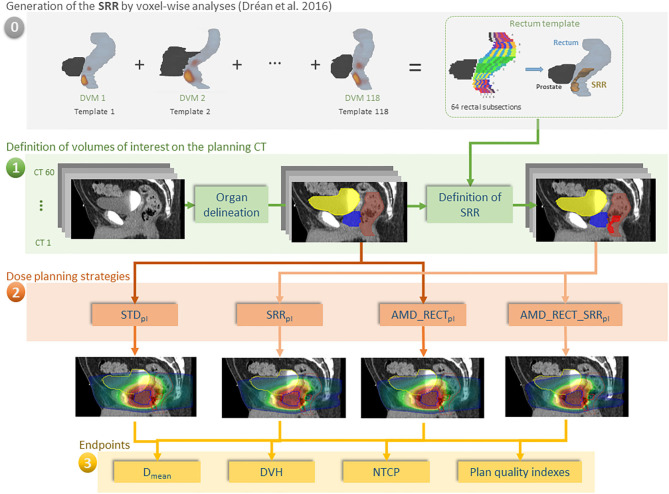
Workflow of our study for 60 individuals. (0) The rectal subregion (SRR) was obtained after averaging results of voxel-wise analysis, using the dose volume maps (DVMs) on 118 different templates in Dréan et al. (10); (1) delineation of OARs and SRR; (2) planning according to four different strategies; (3) endpoint comparisons. In order for the SRR to be generic, the rectum was split in 64 subsections as depicted in Figure A1. The subsections were labeled as belonging to the SRR as a function of the probability (threshold at 50%) of the voxel-wise sub-regions to overlap with the squared subsections. The transfer of the SRR to the TPS was straightforward, following a chess squared mapping of 64 subsections to the 3D volume implemented in RedTox®. Once the organs and sub-regions were segmented, the four planning strategies were implemented, and the dosimetric endpoints were compared.

In the case of prostate cancer IMRT/IGRT, the objectives of this study were to compare four inverse planning strategies: (i) a standard planning (STD_pl_); (ii) a planning with specific SRR constraints without using the AMD model (SRR_pl_); (iii) a strategy using the AMD model applied only to the rectum (AMD_RECT_pl_); (iv) a combined strategy using the AMD model applied to both the rectum and the SRR (AMD_RECT_SRR_pl_). The comparisons were performed via dosimetric, planning quality parameters, and a normal tissue complication probability (NTCP) model. The workflow of the study is depicted in Figure 1.

## Methods and Materials

A total of 60 patients were included in this study. All of them were treated for localized prostate cancer between 2012 and 2015 in the same institution with a volumetric modulated arc therapy (VMAT) technique (1 full-clockwise arc of 18 MV) combined with daily IGRT, using a Synergy/Elekta linac with Agility MLC. A sequential treatment was proposed, delivering first a dose of 50 Gy in 5 weeks to the prostate and seminal vesicles, followed by a boost of 28 Gy in 2.8 weeks to the prostate only. The total number of fractions was therefore 39 fractions, and the dose per fraction was 2 Gy.

### Volumes of Interest on the Planning Computed Tomography

Planning computed tomography (CT) images were acquired on a BigBore (Philips, the Netherlands) scanner, with 2-mm slice thickness. The clinical target volume (CTV) included the prostate and seminal vesicles. The CTV and the OARs (bladder, rectum, and femoral heads) were manually delineated on CT slices according to the French Genitourinary Group (GETUG) recommendations (14, 15). Rectal length was defined as to 2 cm above and below the CTV. The rectal wall was generated with a thickness of 0.5 cm from the external manually delineated rectal contour. The bladder wall was generated with a thickness of 0.7 cm from the external manually delineated bladder contour. PTVs were generated from the CTVs by adding a 0.5-cm margin in all directions.

The definition of the SRR was based on the voxel-wise population study presented in Dréan et al. (10). In summary, repeated voxel-wise analyses (16) were performed on 118 different patients in a leave-one-out scheme. Each patient served iteratively as template of reference to non-rigidly register (17) the remaining 117 patients and propagate the three-dimensional (3D) dose distributions allowing for spatially meaningful dosimetric comparisons. Thus, for each template, voxel-wise analysis (with Wilcoxon test and false discovery rate correction for multiple comparisons) produced a single region where significant dose differences between patients with and without RB appear. These sub-regions were then propagated to a single rectum template, which was divided in 64 subsections, eight sections in both the anteroposterior and axial directions (Figure A1). The generic SRR was finally defined by generating a probability map of presence of the 118 sub-regions within this anatomy. Each one of the 64 subsections with a probability of presence of ≥50% was selected as belonging to the SRR. The rectal sub-region was located close to the prostate (1 cm) and represented 15% of the absolute rectal volume. By construction, the SRR can be easily transferred to a patient-specific anatomy by splitting the rectum in the same 64 subsections, without requiring any registration method. This last step was implemented in an in-house toolbox (RedTox®), which, automatically and in a few seconds, produces a DICOM RTstructure file to be imported in a treatment planning system (TPS) (Figure 1).

Additionally, a volume of the rectum excluding the SRR (“rectum without SRR”) was generated. In the dosimetric study, we considered only the wall for the rectum and the bladder. Thus, we used herein the term *rectum* to refer to the rectal wall; likewise, the term *bladder* to refer to the bladder wall.

### Dose Planning Strategies

Dose planning was performed with Pinnacle v.9.10 (Philips) TPS. The collapsed cone convolution algorithm and a dose grid size of 3 × 3 × 3 mm^3^ were used for dose calculation. Four different VMAT dose planning strategies were applied for each of the 60 patients: STD_pl_, SRR_pl_, AMD_RECT_pl_, and AMD_RECT_SRR_pl_. These four strategies are described below for each planning strategy. The minimum PTV coverage by the 95% prescribed isodose was 95%.

### Standard Planning (STD_pl_)

Treatment plans were generated for each CT according to the GETUG recommendations. GETUG dose–volume constraints were observed throughout: *V*_70Gy_ ≤ 50% and *D*_max_ ≤ 80 Gy for the bladder wall, and *V*_50Gy_ ≤ 50%, *V*_72Gy_ ≤ 25%, and *D*_max_ ≤ 76 Gy for the rectum wall. The main dose constraints used during inverse optimization derived from clinical dose limits and are displayed in Additional Table 1 for the four planning strategies.

### Planning With SRR Constraints (SRR_pl_)

Four SRR dose constraints were applied in addition to STD_pl_ dose constraints: *D*_max_ = 0.8 ^*^Dprescription (weight = 10), *D*15% = 0.75 ^*^Dprescription (weight = 1), *D*25% = 0.60 ^*^Dprescription (weight = 1), and *D*_40%_ = 0.40 ^*^Dprescription (weight = 1).

### Moore's Model and Optimization of Achievable Mean Dose Model

Moore et al. (13) defined a mathematical model allowing for prediction of a radiation plan to achieve the lowest possible mean dose to an OAR overlapping with PTV. This model considers the prescribed dose to the PTV and the overlap volume between the PTV and the considered OAR (13). The general equation of the model was the following:

$$\begin{array}{l} D_{achievable\text{\_}mean}=D_{prescribed}\left(\text{A}+\text{B}\left(\text{C}-e^{-\text{D}xV_{overlap/V_{OAR}}}\right)\right) \end{array}$$

where *V*_overlap_ is the intersection between the PTV and OAR.

This model was created from 17 H&N patients (overlap between parotid glands and PTV) and 8 prostate patients (overlap between rectum and prostate) static IMRT plans, with *A* = 0.2, *B* = 0.8, *C* = 1, *D* = 3. For our study, an adapted Moore's model was fitted based on 15 prostate standard treatment plans from our institution, following the same method as we already published for the H&N case (18). The ratio of the OAR *D*_mean_ to the prescription dose to PTV (*D*_prescribed_) was plotted against the ratio of the overlap volume between PTV and OAR (*V*_overlap_) to the OAR volume (*V*_OAR_), for each patient. Moore's equation coefficients (*A, B, C*, and *D*) were modified by dichotomy, thereby fitting the curve of the model with the lower bound of our local data, which represents the optimal average OAR dose achievable in our selected cohort of patients (*N* = 15).

### Planning With Achievable Mean Dose Model Applied to the Rectum (AMD_RECT_pl_)

The dose constraints used for the standard strategy were applied, as well as additional use of the AMD for the rectum. Our adaptation of the Moore's model provided the following equation for the rectum AMD (AMD_rectum_):

$$\begin{array}{l} AMD_{rectum}=Prescribed\;dose\left(0.22+0.8\left(1-e^{-2V_{overlap}/V_{R}}\right)\right) \end{array}$$

with *V*_overlap_ = overlap between PTV and rectum, and *V*_R_= rectum volume.

### Planning With Achievable Mean Dose Models Applied to the Rectum and the SRR (AMD_RECT_SRR_pl_)

The dose constraints used for the AMD_RECT_pl_ strategy were applied, as well as additional use of the AMD for the SRR. Our adaptation of the Moore's model provided the following equation for the SRR AMD (AMD_SRR_):

$$\begin{array}{l} AMD_{SRR}=Prescribed\;dose(0.34+0.8(1-e^{-2V_{overlap}/V_{SRR}})) \end{array}$$

with *V*_overlap_ = overlap between PTV and SRR, and *V*_SRR_= SRR volume.

## Endpoints and Statistical Analyses

The four planning strategies were evaluated with respect to dosimetric parameters and predicted toxicity endpoints, as well as planning quality indexes.

The dosimetric parameters were as follows: the mean dose for the rectum and for the SRR, and the dose volume histogram (DVH) for the PTV and the OARs. In particular, the following reference RTOG/GETUG points have been reported: volume receiving at least 50 Gy (*V*_50Gy_) and volume receiving at least 70 Gy (*V*_70Gy_) for the SRR and the rectum, *V*_50Gy_ and *V*_70Gy_ for the bladder, and *V*_95%_ for the PTV. The ratio between the *D*_mean_ and the prescribed dose to the PTV (78 Gy) (*D*_mean_ /*D*prescription__PTV_) was also indicated.

The benefit of each strategy to spare the SRR was quantitatively assessed as the difference in the SRR mean dose achieved with respect to the STD_pl_. The risk of toxicity was calculated using the Lyman–Kutcher–Burman NTCP model considering the SRR DVHs. The three parameters (TD50, *n*, and *m*) have been previously identified specifically for the SRR with the maximum likelihood method (19). Thus, our NCTP model predicted the risk of 3-year grade >1 RB with *n* = 0.21, *m* = 0.28, and TD_50_ = 72 (10).

The planning quality parameters were as follows: number of monitor units, irregularity index, and modulation index (20, 21). The irregularity index quantified the non-circularity of the aperture (equal to 1 in case of circular aperture). The modulation index takes into account aperture area and MU number associated to each segment (equal to 0 with a treatment plan without modulation). Conformal and homogeneity indexes were also calculated. The conformal index was defined as the ratio of the volume of PTV receiving 95% of prescribed dose to the volume of PTV. The homogeneity index was defined as the ratio *D*_2%_-*D*_98%_ to the *D*_50%_ of the PTV.

Paired Wilcoxon tests were used to compare the endpoints between the standard planning and each of the three other planning strategies. Correlation tests were used to identify parameters related with the dosimetric benefit of the AMD_RECT_SRR_pl_ strategy. The correlation between the mean dose or the mean dose decrease to the SRR and *V*_SRR_ or *V*_overlap(PTV⋂SRR)_ was tested for each planning strategy. Spearman coefficients (*r*_S_) were computed.

### Ethics Statement

The research has been approved by the institutional review board of the Eugene Marquis Cancer Center, and the patients have been informed of the research.

## Results

### Dosimetric Comparison Between the Four Planning Strategies

**Figure 2:**
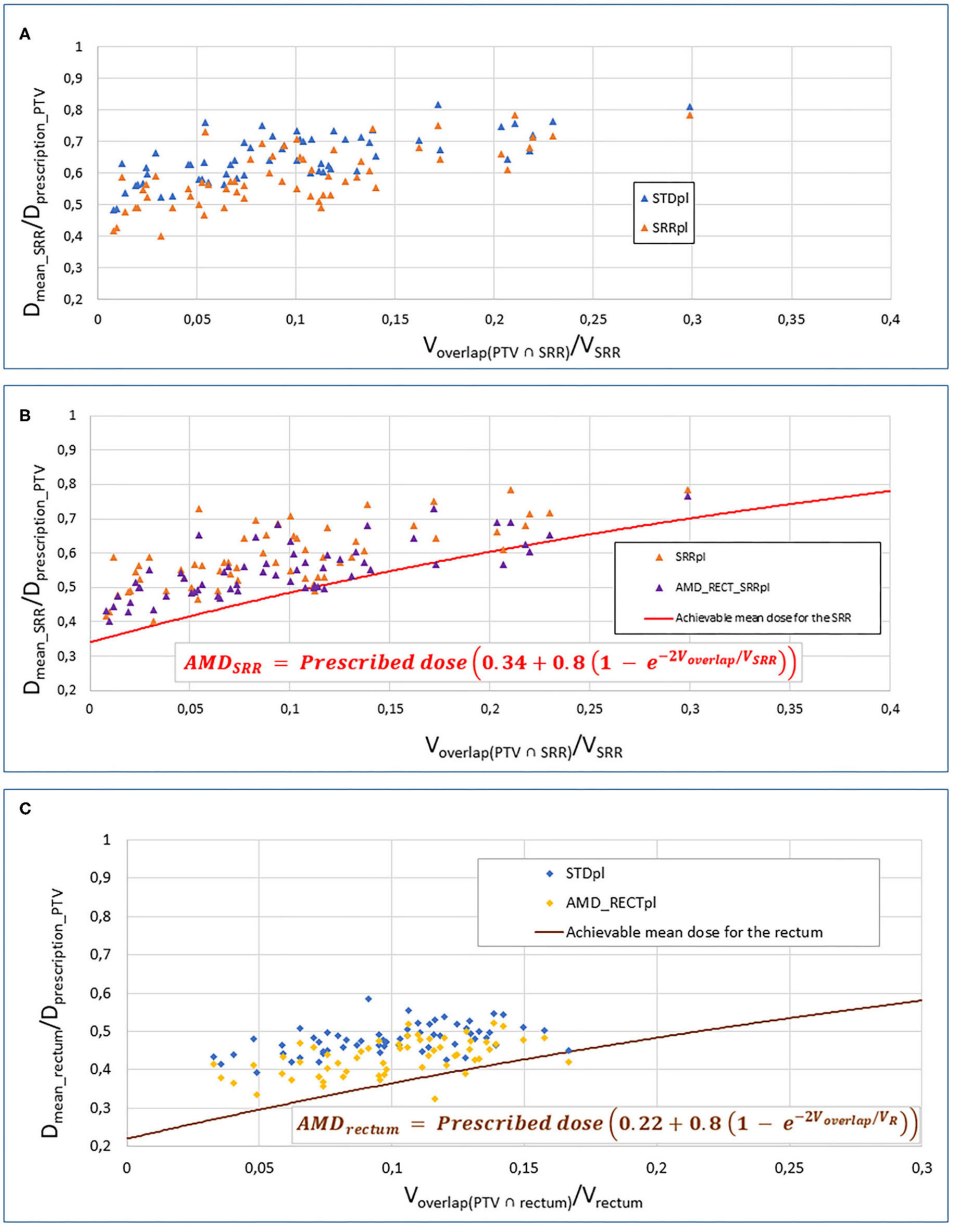
Impact of adding constraints to SRR (SRR_pl_) compared to a STD_pl_ **(A)**, using the AMD model to decrease the mean dose to the SRR (AMD_RECT_SRR_pl_) compared to the SRR_pl_ **(B)** and using the AMD model to decrease the mean dose to the rectum (AMD_RECT_pl_) compared to a STD_pl._ **(C)** AMD, achievable mean dose; STD_pl_, standard planning; SRR_pl_, planning with specific SRR constraints without using AMD model; AMD_RECT_pl_, planning using the AMD model applied to the rectum only; AMD_RECT_SRR_pl_, combined strategy using the AMD model applied to both the rectum and the SRR. The mean doses (*D*_mean_) to the SRR **(A,B)** or rectum **(C)** or “normalized” to the PTV prescribed dose (*D*_mean_rectum_ or SRR/*D*_prescription_PTV_). This ratio is plotted against the percentage of overlap between the SRR **(A,B)** or the rectum **(C)** and the PTV (*V*_overlap(PTV∩*rectum or SRR*)_/*V*_SRR_). The curves display the AMD generated from the equation presented in the figure. The prescription dose to the PTV (*D*prescription__PTV_) was 78 Gy.

Table 1 displays the dosimetric values in the SRR, rectum, the whole rectum without SRR, bladder, and PTV for the four planning strategies. Compared to STD_pl_, AMD_RECT_SRR_pl_ decreased significantly the dosimetric parameters for the rectum (*D*_mean_, *V*_50Gy_, *V*_70Gy_) and for the SRR (*D*_mean_, *V*_50Gy_, *V*_70Gy_), while preserving the PTV coverage. Although the PTV coverage (*V*_95%_) was statistically different between STD_pl_ and SRR_pl_, or AMD_RECT_pl_, the 95% dose coverage constraint was achievable. Compared to STD_pl_, AMD_RECT_pl_ significantly decreased the rectum mean dose from 37.3–33.2 Gy. Compared to STD_pl_, the AMD_RECT_SRR_pl_ strategy significantly decreased the rectum mean dose from 37.3 Gy to 33.7 Gy and the SRR mean dose from 50.6 to 42.9 Gy. Figure 2 shows the impact of adding constraints on SRR (SRR_pl_) compared to a STD_pl_ (Figure 2A), using the AMD model to decrease the mean dose to the SRR (AMD_RECT_SRR_pl_) compared to the SRR_pl_ (Figure 2B) and using the AMD model to decrease the mean dose to the rectum (AMD_RECT_pl_) compared to a STD_pl_ (Figure 2C).

**Table 1:** Volumes, dosimetric endpoints, and NTCP parameters in the subrectal region (SRR), rectum, rectum without SRR, bladder, and PTV by the four planning strategies.

		**Planning strategies**			
		**STD_**PL**_**	**SRR_**PL**_**	**AMD_RE CT_**PL**_**	**AMD_RECT_SRR_**PL**_**
SRR (whole volume)	*D*mean (Gy)	50.6 ± 5.9	45.9 ± 7.1^*^	45.2 ± 5.8^*^	42.9 ± 6.1^*^
	V50_Gy_ (%)	50.4 ± 17.0	40.4 ± 16.7^*^	37.6 ± 14.3^*^	34.6 ± 13.3^*^
	V70_Gy_ (%)	12.8 ± 8.8	10.5 ± 7.1^*^	9.7 ± 7.1^*^	9.7 ± 6.3^*^
	NTCP^#^ (%)	22.8 ± 6.8	19.4 ±6.6^**^	18.4 ± 6.2^**^	17.6 ± 5.7^**^
	Volume (cm^3^) [range]	9.9 ± 4.3 [4.0; 21.3]			
Rectum (wall)	*D*mean (Gy)	37.3 ± 3.0	37.0 ± 3.4	33.2 ± 3.3^*^	33.7 ± 3.6^*^
	V50_Gy_ (%)	29.0 ± 5.3	28.7 ± 6.1	24.3 ± 5.1^*^	25.0 ± 5.6^*^
	V70_Gy_ (%)	12.4 ± 3.3	11.9 ± 3.6^*^	10.4 ± 3.1^*^	10.8 ± 3.4^*^
	Volume (cm^3^) [range]	32.4 ± 7.8 [48.7; 18.4]			
Rectum volume without SRR (whole volume)	*D*mean (Gy)	34.6 ± 3.7	34.6 ± 4.0	30.0 ± 3.8^*^	30.5 ± 4.2^*^
	V50_Gy_ (%)	23.0 ± 6.8	23.5 ± 7.5	17.5 ± 6.0^*^	18.5 ± 6.4^*^
	V70_Gy_ (%)	7.2 ± 2.5	6.5 ± 2.5^*^	5.7 ± 2.2^*^	5.5 ± 2.2^*^
	Volume (cm^3^) [range]	59.8 ± 24.5 [23.9; 113.9]			
Bladder (wall)	V50_Gy_ (%)	30.4 ± 12.4	29.8 ± 12.0^*^	29.7 ± 12.0^*^	29.4 ± 12.5^*^
	V70_Gy_ (%)	14.2 ± 6.1	14.0 ± 6.0	14.2 ± 6.0	14.2 ± 6.2
	Volume (cm^3^) [range]	67.8 ± 26.8 [124.3; 24.4]			
PTV	D95% (%)	96.2 ± 0.6	95.8 ± 0.5^*^	96.5 ± 0.5^*^	96.1 ± 0.7
	Homogen eity index	0.08 ± 0.01	0.10 ± 0.01^*^	0.08 ± 0.01^*^	0.10 ± 0.02^*^
	Conforma l index	0.98 ± 0.01	0.97 ± 0.01^*^	0.98 ± 0.01	0.97 ± 0.02^*^
	Volume (cm^3^) [range]	109.7 ± 36.1 [41.4; 260.3]			
Overlap between SRR and PTV	Volume (cm^3^) [range]	0.9 ± 0.5 [0.1; 2.4]			
Overlap between rectum and PTV	Volume (cm^3^) [range]	3.2 ± 1.3 [0.9; 6.9]			

*AMD, achievable mean dose; STD_pl_, standard planning; SRR_pl_, planning with specific SRR constraints without using AMD model; AMD_RECT_pl_, planning using the AMD model applied to the rectum only; AMD_RECT_SRR_pl_, combined strategy using the AMD model applied to both the rectum and the SRR*.

*Values are mean ± standard deviation*.

*The NTCP^#^ values have been calculated by using the following parameters: n = 0.21, m = 0.28, and TD_50_ = 72. The NTCP evaluates the risk of 3-year grade ≥1 rectal bleeding (10)*.

* *p < 0.05 (assuming significance level) of the Wilcoxon test comparing the standard strategy (STD_pl_) to each of the tested strategy*.

** *p < 0.001*.

**Figure 3:**
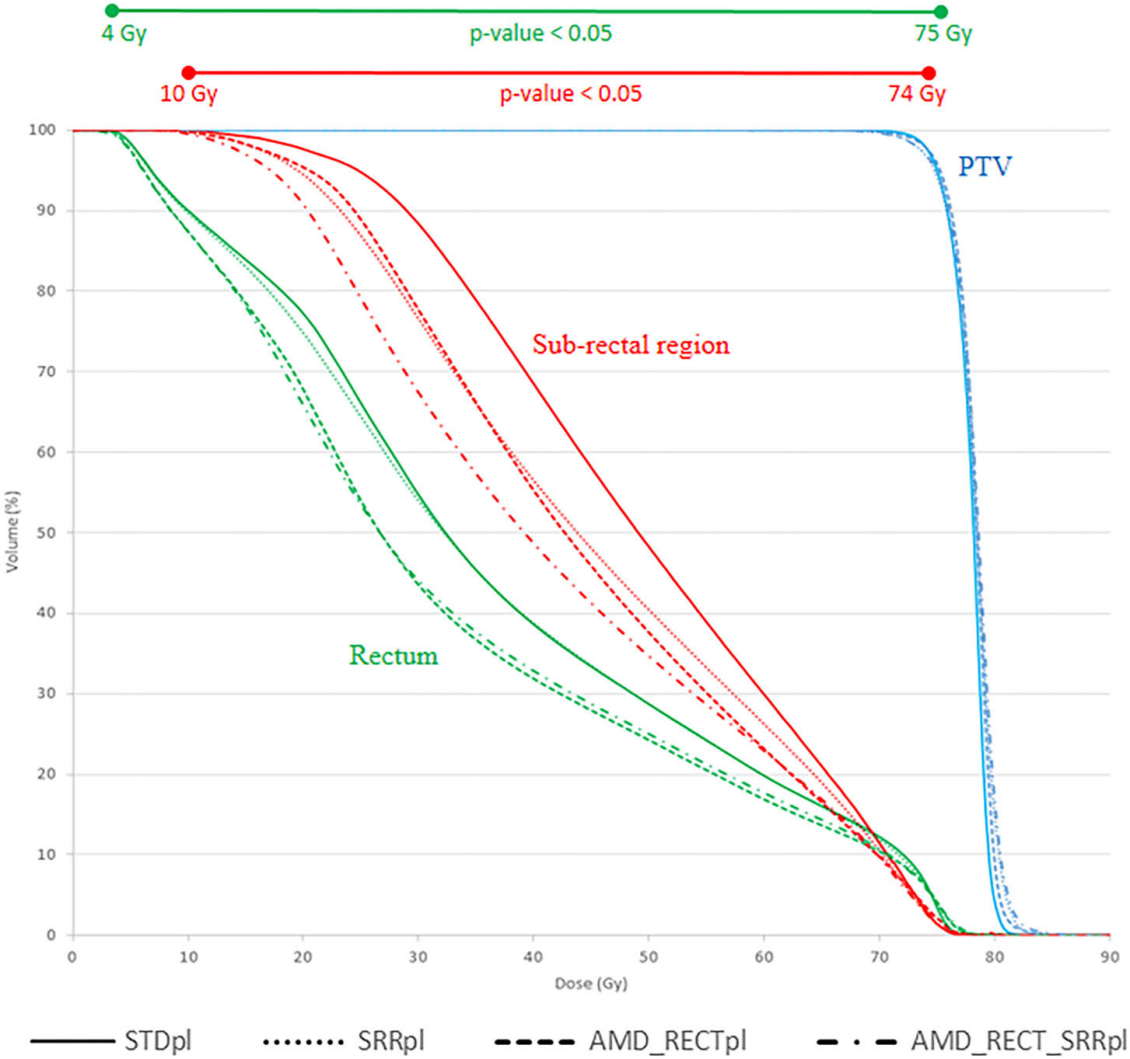
Mean DVH corresponding to each of the four planning strategies. AMD, achievable mean dose; STD_pl_, standard planning; SRR_pl_, planning with specific SRR constraints without using AMD model; AMD_RECT_pl_, planning using the AMD model applied to the rectum only; AMD_RECT_SRR_pl_, combined strategy using the AMD model applied to both the rectum and the SRR. The prescription dose was 78 Gy. Wilcoxon tests were used to compare the DVHs from the standard strategy (STD_pl_) to those of each planning strategy. Significant differences (*p* < 0.05) are displayed at the top of the graphic.

Figure 3 shows the average DVH of the PTV, rectum, and SRR for the four strategies. Compared to STD_pl_, AMD_RECT_SRR_pl_ reduced significantly the volume of the rectum and the SRR receiving a dose between 4 and 75 Gy and between 10 and 74 Gy, respectively.

**Figure 4:**
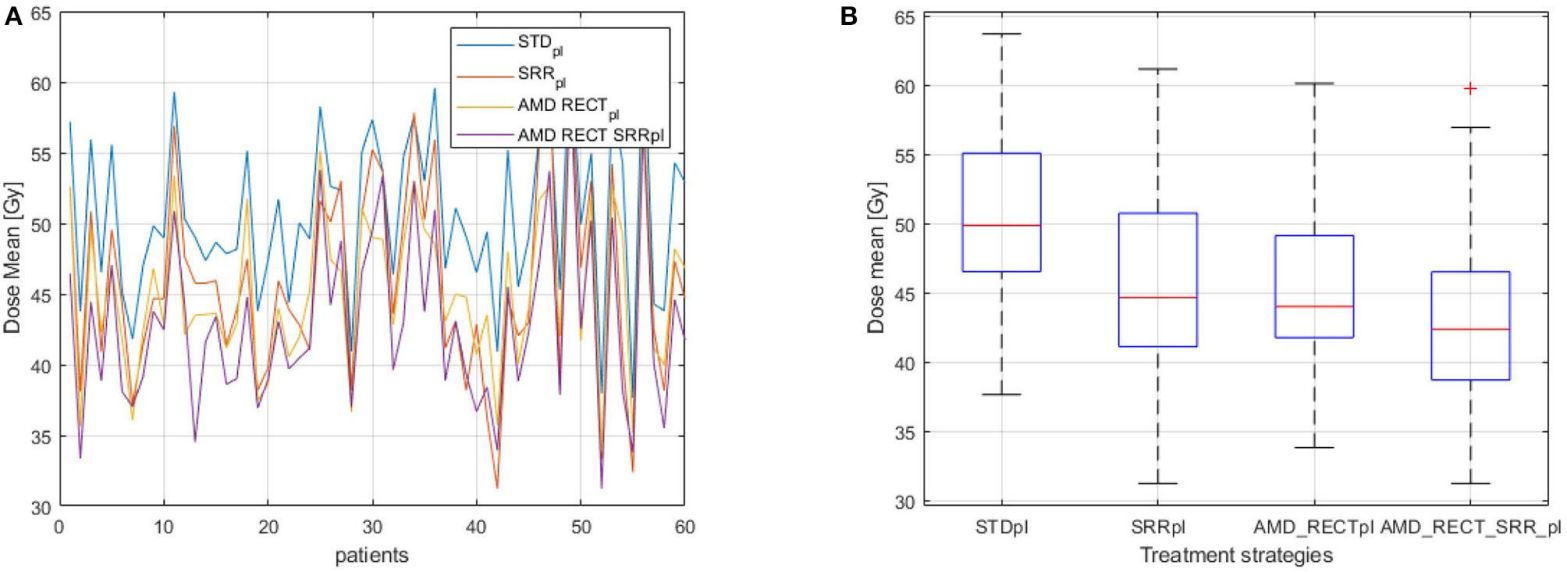
**(A)** *D*_mean_ to the SRR for the 60 patients; **(B)** Distribution of *D*_mean_ to the SRR according to the planning strategies. Mean dose (*D*_mean_) to the subrectal region (SRR) for the four planning strategies (STD_pl_, SRR_pl_, AMD_RECT_pl_, and AMD_RECT_SRR_pl_). AMD, achievable mean dose; STD_pl_, standard planning; SRR_pl_, planning with specific SRR constraints without using the AMD model; AMD_RECT_pl_, planning using the AMD model applied to the rectum only; AMD_RECT_SRR_pl_, combined strategy using the AMD model applied to both the rectum and the SRR.

**Figure 5:**
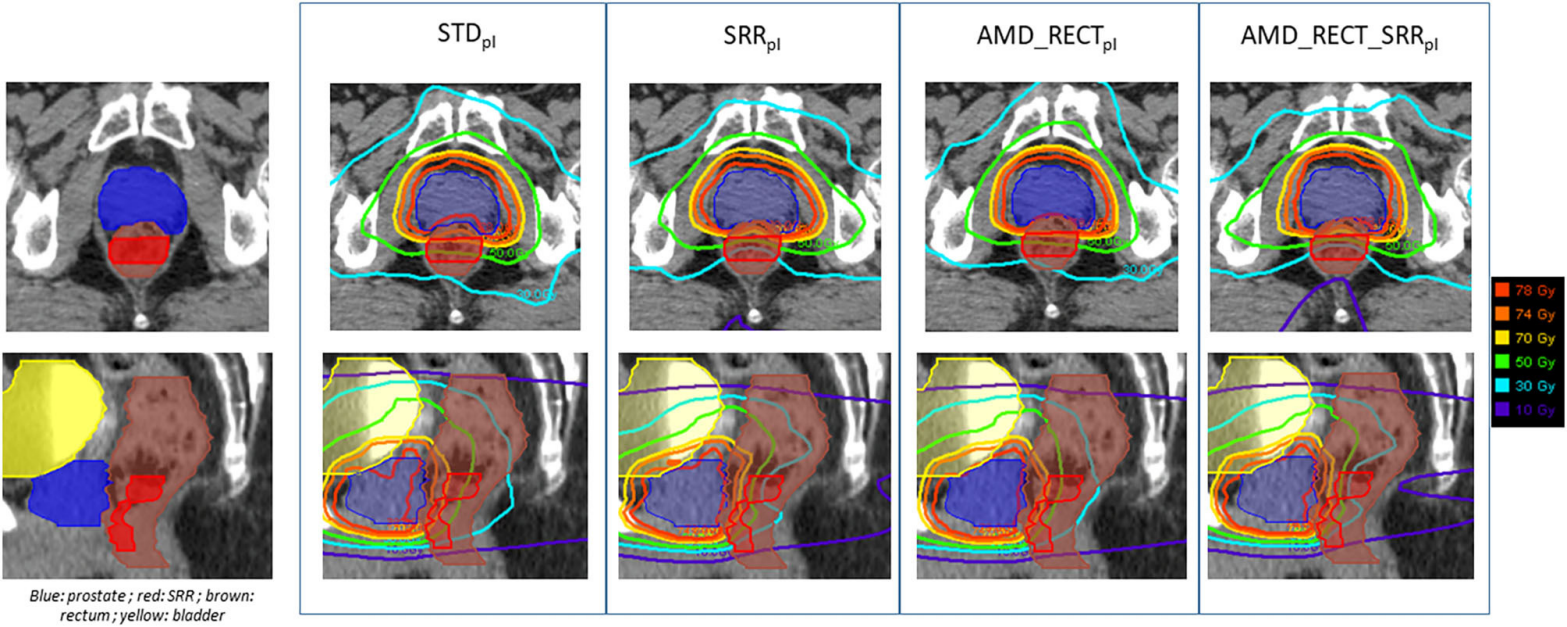
Illustration of the dose distributions corresponding to each of the four planning strategies for a given patient. AMD, achievable mean dose; STD_pl_, standard planning; SRR_pl_, planning with specific SRR constraints without using the AMD model; AMD_RECT_pl_, planning using the AMD model applied to the rectum only; AMD_RECT_SRR_pl_, combined strategy using the AMD model applied to both the rectum and the SRR. The prescribed dose is 78 Gy.

Figure 4 depicts the mean dose to the SRR for each planning strategy (Figures 4A,B) to the SRR between STD_pl_ and each tested planning (SRR_pl_, AMD_RECT_pl_, and AMD_RECT_SRR_pl_). Compared to STD_pl_, AMD_RECT_SRR_pl_ decreased the mean dose to the SRR up to 16.2 Gy. The median SRR mean dose reduction, compared to STD_pl_, was 4.6 Gy when using SRR_pl_, 5.1 Gy when using AMD_RECT_pl_, and 7.9 Gy when using AMD_RECT_SRR_pl_. Figure 5 illustrates the dose distribution corresponding to each of the four planning strategies for a given patient.

No correlation (|*r*_S_| < 0.21) was found between the mean dose or the mean dose decrease to the SRR and *V*SRR or the *V*overlap(PTV _n_ SRR).

### NTCP Comparison Between the Four Strategies

Table 1 displays the NCTP values computed from the SRR when considering the four planning strategies. Compared to STD_pl_, the AMD_RECT_SRR_pl_ strategy significantly decreased the estimated risk of RB. The NTCP values decreased from 22.8 to 17.6% when considering the SRR DVH.

### Quality Comparison of the Planning Between the Four Strategies

Table 1 displays the homogeneity and conformal indexes in the PTV by the four planning strategies. The homogeneity index significantly increased for AMD_RECT_SRR_pl_ with respect to STD_pl_. The conformal index significantly decreased for the AMD_RECT_SRR_pl_ with respect to STD_pl_. Table 2 displays the planning quality parameters for each strategy. The MU significantly increased from 372 for STD_pl_ to 454 MU for AMD_RECT_SRR_pl_. The irregularity and the modulation indexes significantly increased for the AMD_RECT_SRR_pl_ with respect to STD_pl_.

**Table 2:** Planning quality parameters for each planning strategy.

**Planning optimization**	**STD_**PL**_**	**SRR_**PL**_**	**AMD_RECT_**PL**_**	**AMD_RECT_SRR_**PL**_**
Monitor units (MU)	372 ± 25	396 ± 29^*^	441 ± 35^*^	454 ± 42^*^
Irregularity index (ideal value → 1)	4.35 ± 1.25	5.24 ± 1.08^*^	5.92 ± 1.62^*^	6.86 ± 1.8^*^
Modulation index (ideal value → 0)	0.63 ± 0.04	0.65 ± 0.04^*^	0.69 ± 0.03^*^	0.69 ± 0.04^*^

*AMD, achievable mean dose; STD_pl_, standard planning; SRR_pl_, planning with specific SRR constraints without using AMD model; AMD_RECT_pl_, planning using the AMD model applied to the rectum only; AMD_RECT_SRR_pl_, combined strategy using the AMD model applied to both the rectum and the SRR*.

*Values are mean ± standard deviation*.

* *p < 0.05 (assuming significance level) of the Wilcoxon test comparing the standard strategy to each of the tested strategy*.

## Discussion

This paper proposed a methodology for decreasing rectal toxicity by adding a patient-specific sub-region in the prostate inverse radiotherapy planning and by using a specific achievable mean dose model. We compared four different inverse planning strategies in terms of dosimetric benefit, planning quality parameters, and NTCP prediction.

A considerable dose reduction can be achieved to both the rectum and the SRR with the combined approach (AMD_RECT_SRR_pl_) compared to the standard planning (Table 1). This planning strategy appears particularly appealing because it is not invasive, can be easily customized, does not increase the treatment workload, and offers improved OAR sparing while preserving PTV coverage. Furthermore, the application of the NTCP model ratifies a reduction in rectal toxicity. It must be pointed out that decreasing the dose in the SRR via the SRR_pl_, did not have any impact on dose increase elsewhere in the rectum (Table 1, rectum without SRR). On the contrary, a diminution on SRR mean dose was accompanied by a global diminution of dose in the rectum (*D*_mean_ and *V*_70Gy_).

For the dose planning step, the rectal and bladder walls were used in order to meet the French GETUG recommendations. However, both the whole 3D organ and the wall can be considered either for planning dose constraints or for toxicity prediction. As shown in the literature and confirmed in the clinical routine, the recta DVH and rectal wall DVHs are highly correlated (22). Moreover, rectal wall DVH provides a moderate improvement when fitting NTCP models (23).

Concerning the dose constraints in the TPS, we implemented a version of the AMD model proposed by Moore et al. (13) to decrease the dose in the rectum and the SRR. This model was first introduced in a dosimetric quality control context with the aim of improving the planner's experience in the case of inverse planning. They proposed a simple tool using a generic model to predict OAR mean dose taking into account the PTV and OAR volume overlap. Their results showed a significant reduction to the mean dose for both rectum and parotid glands, compared to a standard planning approach. However, the application of the AMD model requires a customization to each clinical center and to each tumor location, as shown by Powis et al. (24) and Delaby et al. (18). Powis et al. (24) improved plan quality for prostate cancer. With their customized AMD model, the rectum mean dose was significantly decreased from 41.6 to 36 Gy, for a prescribed dose of 74 Gy to the prostate. Delaby et al. (18) showed a dose reduction of 6.1 Gy to the parotid glands using their own adaptation of AMD model for H&N. In our study, the dosimetric benefit on rectal mean dose was 4.1 Gy (Table 1, STD_pl_ minus AMD_RECT_pl_) for a prescribed dose of 78 Gy to the prostate.

Voxel-wise analysis by non-rigid registration has become a well-established methodology able to unveil the likely heterogeneous radiosensitivity across the organs, which may be helpful in the identification of sub-regions to be spared at the planning step. One of the major advantages of the voxel-wise analysis is its ability to explore the full 3D anatomy without prior assumptions regarding the location of regions correlating with toxicity (25). As compared with dose surface maps, which have also been used for this purpose, the voxel-based methods present the advantage of generating 3D volumes that can be transferred to the clinical practice in a straightforward way.

The rectal SRR, considered in this study, was previously identified through voxel-based analysis as predictive of RB in a series of 118 prostate cancer patients treated with IMRT/IGRT and validated on a testing data set of 53 patients (10). This SRR represented the 15% of the absolute rectal volume and was located in the inferior–anterior rectal region. If the benefit of using the SRR was shown in the previous study for toxicity prediction (10), the present work additionally explores the potential advantage of sparing this SRR during the planning. The same workflow can be applied in other locations such as lung, bladder, or H&N, where sub-regions have been previously identified.

Our study presents several limitations. The main issue is the lack of clinical data to demonstrate the real improvement of combining the SRR with the AMD strategies in toxicity reduction. Furthermore, we were not able to correlate geometric characteristics (overlap volume between the PTV and SRR) with the dose reduction within the SRR. As mentioned before, one of the issues that may arise in voxel-wise analysis, stemming from the interindividual variability, is the reproducibility of the SRRs in different templates and the reliability of non-rigid registration (26). The SRR used in this study was, however, previously generated through repeated voxel-wise analyses on 118 different templates in a leave-one-out strategy (10) using a validated non-rigid registration method (17), confirming the robustness to the computed SRR. Because of the deformable nature of organs and soft tissues, another potential issue is that the planning dose may not be representative of the true delivered dose. Indeed, considering the mean dose to the rectum, the dose difference between the planned dose and the estimated cumulated dose by elastic registration has been quantified to be around 2 Gy (27). Because organs are moving and deforming between fractions, new models should include this information either by quantifying daily deformations with MVCT (28) or by estimating cumulative dose with statistical methods as in Rios et al. (29). Other image modalities such as cone-beam CT or magnetic resonance imaging can also provide daily images helping to quantify anatomical changes.

Although very useful for indicating achievable doses, the use of the AMD model presents geometric and dosimetric limitations. For instance, it only considers the global overlap (OAR ⋂ PTV) volume, without taking into account the OAR shape, orientation, or geometric irregularities. Wu et al. (30) pointed out this issue by showing similar OAR ⋂ PTV overlap configurations but with different OAR shapes. Hence, two different configurations would yield equivalent AMD. They also introduced the concept of overlap volume histogram (OVH) to describe the fractional volume of the structure of an OAR but with respect to a specified distance to the target volume. The OVH is a shape relationship descriptor, measuring the proximity of the OAR to the target, which also provides a way to infer the likely DVH of an OAR. A relation between DVH and OVH could be computed near the target volume in order to refine the achievable DVH. Wall et al. (31) investigated DVH-OVH (rectum and bladder volumes) correlations in a series of 124 prostate patients. By replanning 31 randomly selected patients, the rectum mean dose decreased by 9.4 Gy, compared to the initial planning. Another limitation of the AMD model is the use of the mean dose as a constraint. The mean dose is rarely considered for dose–toxicity prediction in the rectum as are rather the higher doses, which are correlated to rectal toxicity (27).

A practical limitation also exists regarding the generalization of the proposed workflow in a clinical setup. This stems from the fact that transferring the SRR to a specific patient anatomy requires the use of nonintegrated tools in the TPS for the time being. Nevertheless, the algorithm for the SRR generation (10) has been implemented in an in-house toolbox (RedTox®), which produces a DICOM-RT structure of the SRR in a few seconds, which can be imported within any TPS. This workflow provides therefore a way forward on the implementation of personalized treatments.

## Conclusion

In case of prostate cancer radiotherapy, a sub-region highly predictive of RB, determined by voxel-wise analyses, was transferred to patient-specific anatomies for dose planning. The integration of this SRR into the TPS allows tailoring a personalized planning with dose constraints based on an AMD model. Compared to the standard planning approach, the proposed AMD strategy decreases the rectal and the SRR mean doses by 3.6 and 7.7 Gy, respectively, while preserving PTV coverage. This dosimetric benefit may be translated into a relative reduction in probability of RB by 22%. Following this workflow, a reduced-toxicity personalized treatment can be achieved. Nevertheless, such clinical benefit on IMRT/IGRT needs to be confirmed in prospective clinical trials.

## Data Availability Statement

The datasets generated for this study are available on request to the corresponding author.

## Author Contributions

All authors listed have made a substantial, direct and intellectual contribution to the work, and approved it for publication.

## Conflict of Interest

The authors declare that the research was conducted in the absence of any commercial or financial relationships that could be construed as a potential conflict of interest. The handling editor declared a past co-authorship with several of the authors EM, OA, and RC.
