# Supplementary material for: Planning With Patient-Specific Rectal Sub-Region Constraints Decreases Probability of Toxicity in Prostate Cancer Radiotherapy
Source: Front Oncol. 2020 Sep 11;10:1597. doi: 10.3389/fonc.2020.01597 (PMC7517942; doi:10.3389/fonc.2020.01597)
Supplement: Supplementary file 1 [file Data_Sheet_1.pdf]

Planning with patient-specific rectal subregion constraints decreases probability of toxicity in prostate cancer radiotherapy

Caroline Lafond[1], Anaïs Barateau[1], Joël N'Guessan[1], Nicolas Perichon[1], Nolwenn Delaby[1], Antoine Simon[1], Pascal Haigron[1], Eugenia Mylona[1]; Oscar Acosta[1], Renaud de Crevoisier[1]

1. Univ Rennes, CLCC Eugène Marquis, INSERM, LTSI - UMR 1099, F-35000 Rennes, France

Corresponding author: Oscar Acosta

Hosting Journal/Specialty: **Frontiers in Oncology** - section **Radiation Oncology**

Research Topic: **Modeling for Prediction of Radiation-Induced Toxicity to Improve Therapeutic Ratio in the Modern Radiation Therapy Era**

Acknowledgments

This work was partially supported by the French Institut National of Cancer for the STIC-IGRT-P, and by the French National Research Agency (ANR) in the framework of the "Investing for the future" Program through Labex CAMI (ANR-11- LABX-0004) and Labex CominLabs ANR-10-LABX-07-01

Additional Figures/Tables

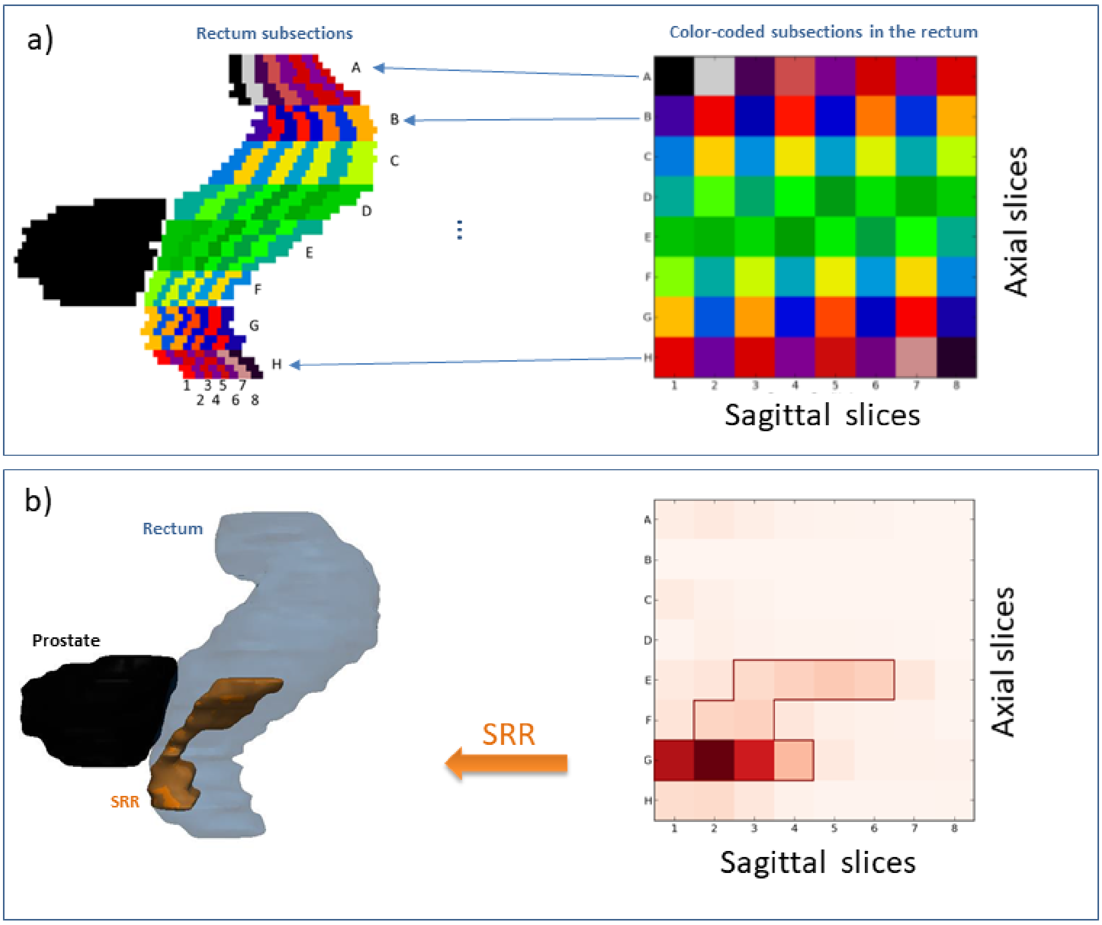

Figure A1. Construction of rectal subdivision in order to obtain the sub-rectal region (SRR)

a) The rectum was divided in 64 subsections, eight sections in both the anteroposterior and axial directions. The color code illustrates the mapping between a chess cartography and the 3D volume. Each square represents a portion of the rectum. All the 118 templates were mapped in the same way. b) The probability maps were obtained in this cartography after voxel wise analyses repeated 118 times. The portions with the highest probability (50%) are mapped back to the 3D volume yielding the SRR. To transfer to a new patient, the rectum is split in the same way and the SRR is propagated by labelling specific portions (Toolbox RedTox).

	Volume of interest	Dose constraint	**Weight**
**STD_{pl}**	PTV	$D_{min} = 0.99*D_{prescription}$<br>$D_{uniform} = 1*D_{prescription}$<br>$D_{max} = 1.01*D_{prescription}$	100<br>100<br>100
	Rectum	$D_{5\%} = 0.825*D_{prescription}$<br>$D_{15\%} = 0.6*D_{prescription}$<br>$D_{30\%} = 0.39*D_{prescription}$<br>$D_{max} = 0.9*D_{prescription}$	2<br>2<br>2<br>100
	Bladder	$D_{max} = 0.935*D_{prescription}$	100
	Femoral heads	$D_{5\%} = 0.435*D_{prescription}$	1
	Optimization shell from 2 mm to 7 mm of the PTV	$D_{max} = 0.95*D_{prescription}$	50
	Optimization shell from 7 mm to 12 mm of the PTV	$D_{max} = 0.9*D_{prescription}$	50
	Optimization shell from 20 mm of the PTV to external contour	$D_{max} = 0.50*D_{prescription}$	10
**SRR_{pl}**	Dose constraints of STD_{pl}		
	SRR excluding PTV	$D_{max} = 0,9*D_{prescription}$	100
		$D_{15\%} = 0.83*D_{prescription}$	1
		$D_{25\%} = 0.6*D_{prescription}$	1
		$D_{40\%} = 0.4*D_{prescription}$	1
	SRR including PTV	$D_{uniform} = 0.96*D_{prescription}$	80
**AMD_RECT_{pl}**	PTV	$D_{min} = 0.99*D_{prescription}$<br>$D_{uniform} = 1*D_{prescription}$<br>$D_{max} = 1.01*D_{prescription}$	100<br>100<br>100
	Rectum	$D_{5\%} = 0.82*D_{prescription}$<br>$D_{15\%} = 0.5*D_{prescription}$<br>$D_{30\%} = 0.38*D_{prescription}$<br>$D_{max} = 0.9*D_{prescription}$	5<br>5<br>5<br>90
	Bladder	$D_{max} = 0.935*D_{prescription}$	100
	Femoral heads	$D_{5\%} = 0.435*D_{prescription}$	1
	Optimization shell from 2 mm to 7 mm of the PTV	$D_{max} = 0.95*D_{prescription}$	50
	Optimization shell from 7 mm to 12 mm of the PTV	$D_{max} = 0.9*D_{prescription}$	50

	Optimization shell from 20 mm of the PTV to external contour	D_{max} = 0.50*$D_{prescription}$	10
**AMD_RECT_SRR$_{pl}$**	Dose constraints of AMD_RECT$_{pl}$		
	SRR excluding PTV	D_{max} = 0,8*$D_{prescription}$	90
		$D_{15\%}$ = 0.76*$D_{prescription}$	3
		$D_{25\%}$ = 0.58*$D_{prescription}$	3
		$D_{40\%}$ = 0.38*$D_{prescription}$	3
	SRR including PTV	$D_{uniform}$ = 0.95*$D_{prescription}$	80

Additional Table 1. Dose constraints for inverse optimization

AMD: achievable mean dose; STD$_{pl}$: standard planning; SRR$_{pl}$: planning with specific SRR constraints without using AMD model; AMD_RECT$_{pl}$: planning using the AMD model applied to the rectum only; AMD_RECT_SRR$_{pl}$: combined strategy using the AMD model applied to both the rectum and the SRR.
